# Evaluation of ferroptosis markers and laboratory findings in patients with juvenile idiopathic arthritis

**DOI:** 10.1590/1806-9282.20251637

**Published:** 2026-06-19

**Authors:** Seyda Dogantan, Burcu Bozkaya Yücel, Peren Perk, Arzu Şekerci Yüksel, Rahime Koç, Adem Keskin

**Affiliations:** 1 Başakşehir Çam ve Sakura City Hospital, Department of Pediatric Rheumatology – Istanbul, Turkey.; 2 Samsun Research and Education Hospital, Department of Pediatric Rheumatology – Samsun, Turkey.; 3 Başakşehir Çam ve Sakura City Hospital, Department of Pediatric Neurology – Istanbul, Türkiye.; 4 Başakşehir Çam ve Sakura City Hospital, Department of Medical Biochemistry – İstanbul, Türkiye.; 5 Aydın Adnan Menderes University, Faculty of Medicine, Department of Medical Biochemistry – Aydin, Turkey.

**Keywords:** Juvenile idiopathic arthritis, Ferroptosis, Malondialdehyde, Methotrexate, ELISA

## Abstract

**OBJECTIVE:**

Juvenile idiopathic arthritis is the most prevalent chronic rheumatic disease of childhood. Ferroptosis is a distinct, regulated form of cell death that is iron-dependent and driven by the accumulation of lipid peroxides on cell membranes. The aim of this study was to measure the soluble transferrin receptor and malondialdehyde levels as ferroptosis markers in Turkish children with Juvenile idiopathic arthritis. We also evaluated the laboratory findings in the patients.

**METHODS:**

Thirty children diagnosed with Juvenile idiopathic arthritis and 30 healthy children were included in the study. The sTfR1 and the malondialdehyde levels were measured by enzyme-linked immunosorbent assay.

**RESULTS:**

The mean age of the patient group was 10.13±3.98, and the mean age of the control group was 10.67±3.21. Blood soluble transferrin receptor and malondialdehyde levels did not differ between the patient and control groups (p>0.05). In contrast, the p-value for the malondialdehyde results was statistically borderline. In the patient group, sedimentation rates were found to correlate with serum amyloid A, C-reactive protein levels. In addition, the highest malondialdehyde levels were found in systemic Juvenile idiopathic arthritis, and the highest soluble transferrin receptor levels were found in psoriatic arthritis subtypes. Both malondialdehyde and soluble transferrin receptor levels were lowest in those treated with methotrexate.

**CONCLUSION:**

Although our findings show that soluble transferrin receptor and malondialdehyde levels did not differ between patient and control groups, larger-scale studies are needed to evaluate more specific biomarkers and/or targeted tissue sampling in different subtypes, controlling for confounding factors such as treatment. This could open new horizons for therapies targeting ferroptosis.

## INTRODUCTION

Juvenile idiopathic arthritis (JIA), which usually appears before the age of 16, is the most common rheumatic connective tissue disease of childhood. Characterized by leukocyte infiltration in the synovial fluid of the joints, this disease presents with chronic inflammation lasting at least 6 weeks and can lead to progressive damage to the joint tissue^1^. While dysregulation of the adaptive immune system primarily contributes to the pathophysiology in the oligoarticular and polyarticular subtypes of JIA, dysregulation of the innate immune system appears more prominent in the systemic form. This is particularly associated with increased levels of interleukin (IL)-1β, IL-6, and IL-18. Activation of monocytes and neutrophils, the key effector cells of joint inflammation and destruction, is one of the common pathogenic mechanisms in JIA^2^. In addition to the immune response, oxidative stress also plays a significant role in the pathogenesis of the disease. Oxidative stress results from an imbalance between the increase in reactive oxygen species (ROS) and reactive nitrogen species and the detoxification of these reactive intermediates or the repair of the protein, lipid, and DNA damage they cause^2^.

In recent years, ferroptosis, a novel type of programmed cell death triggered by iron-dependent lipid peroxidation, has been identified. It is characterized by mitochondrial atrophy, increased membrane density, and changes in mitochondrial morphology^3^. An imbalance between oxidant and antioxidant systems, resulting from abnormal expression and activity of redox-active enzymes, can trigger ferroptotic cell death^4^. Ferroptosis; It is characterized by high lipid hydroperoxide levels and iron loading, and is a caspase and necrosome-independent cell death mechanism^5^.

Increasing experimental evidence suggests that excessive iron accumulation can lead to oxidative tissue damage and organ dysfunction, contributing to the development of various diseases such as diabetes, cardiomyopathy, and cirrhosis. Karim and colleagues reported that excess iron disrupts cellular iron homeostasis, negatively impacts chondrocyte function, and leads to oxidative stress and cell death^6^. Furthermore, it has been shown that excess iron can exacerbate joint damage in osteoarthritic joints through increased ROS production and the accumulation of calcium phosphate, hydroxyapatite, and/or uric acid crystals^7^.

Soluble transferrin receptor (sTfR) is the circulating form of transferrin receptor 1 (TfR1) found on the cell surface and plays a role in transporting iron into cells by binding to transferrin, the main iron-carrying protein in the blood^8^. On the other hand, malondialdehyde (MDA) is one of the most common and reliable biomarkers of lipid peroxidation and is frequently used in the assessment of oxidative stress.

In this study, we aimed to evaluate serum sTfR and MDA levels as markers associated with ferroptosis in Turkish children diagnosed with JIA. We also analyzed the patients’ laboratory findings and examined the relationships between these parameters.

## METHODS

### Study design

The patient group consisted of 30 patients diagnosed with JIA followed up at the Pediatric Rheumatology Department of Başakşehir Çam and Sakura City Hospital. JIA diagnosis was made according to the International Union of Rheumatology Societies (ILAR) classification criteria^9^. Definitive diagnosis was confirmed in the presence of two primary criteria or one primary and two secondary criteria. None of the patients had additional chronic diseases. Clinical and laboratory findings of all patients were systematically evaluated. The control group consisted of 30 healthy children from the same geographic region, matched for age and gender, and without known chronic diseases.

In accordance with the ethical principles of the Helsinki Declaration, written informed consent was obtained from all participants and/or their legal guardians before inclusion in the study. Ethical approval for the study was granted by the Ethics Committee of Başakşehir Çam and Sakura City Hospital (Approval No: KAEK-11/10.09.2025.334).

### Soluble transferrin receptor and malondialdehyde analysis

The sTfR and the MDA levels were measured by enzyme-linked immunosorbent assay (ELISA). These assessments were performed using the Sunred 201-12-7968 Human Soluble Transferrin Receptor ELISA kit and the Sunred 201-12-1952 Human MDA ELISA kit, respectively.

### Statistical analysis

For statistical analyses, International Business Machines Statistical Package for the Social Sciences Statistics 22 (IBM Corp., Armonk, NY, USA) software was used for Windows. The normality of continuous variables was assessed using the Shapiro-Wilk test. It was determined that the data did not conform to a normal distribution. Therefore, continuous variables were expressed as median [25th percentile (Q1)–75th percentile (Q3)]. The Mann-Whitney U test was used for intergroup comparisons, and the chi-square test was used for comparisons of categorical variables. The Kruskal-Wallis test was used for comparisons of subgroups. Spearman correlation analysis was used to evaluate the relationship between variables. Bonferroni correction was applied for multiple comparisons. A statistical significance level of p<0.05 was accepted for all analyses.

## RESULTS

Thirty patients aged 2–17 years, diagnosed with JIA, and 30 healthy children aged 6–17 years were included in the study. The mean age of the patient group was 10.13±3.98, and the mean age of the control group was 10.67±3.21. Of the patient group, 14 were women (46.67%) and 16 were men (53.33%). In the control group, these rates were 10 (33.33%) and 20 (67.67%), respectively. No significant difference was found between the two groups in terms of mean age and gender ratios (p=0.570 and p=0.292, respectively). Laboratory findings for the patient group are presented in Table 1. Blood sTfR and MDA levels did not differ between the patient and control groups. Erythrocyte sedimentation rate (ESR) and creatinine levels were found to be higher in the patient group than in the control group.

**Table 1:** Comparison of postoperative step counts and gastrointestinal outcome between groups.

	Patient group (n=30)	Control group (n=30)	p
MDA	12.25 (9.45–17.44)	16.81 (12.8–22.87)	0.052
STfR	0.6 (0.55–0.73)	0.68 (0.51–0.97)	0.203
Serum amyloid A	0.56 (0.29–7.11)	0.65 (0.33–1.33)	0.749
Sedimentation	9 (4–20)	2.5 (1–4)	**<0.001**
CRP	0.5 (0.3–6.1)	1.2 (0.3–2.1)	0.976
Leucocyte	7.27 (6.06–8.56)	7.37 (6.1–9.19)	0.767
Hematocrit	36.4 (35–38.4)	38.5 (34.2–42.9)	0.109
Hemoglobin	12.1 (11.6–12.5)	12.6 (11–13.7)	0.437
Neutrophile	3.59 (2.58–5.4)	3.48 (2.78–4.06)	0.929
Lymphocyte	2.65 (2.21–3.38)	2.98 (2.49–3.6)	0.203
PLT	309 (245–369)	327.5 (287–363)	0.673
MCV	78 (74.7–80.1)	78.9 (77–82.8)	0.367
BUN	22.4 (18.2–28)	24 (22–26)	0.135
Creatinine	0.52 (0.42–0.64)	0.28 (0.28–0.28)	**<0.001**
ALT	15.5 (10–18)	14.5 (12–17)	0.923
AST	23 (19–28)	20 (17–28)	0.207
LDH	216 (193–257)	210 (182–240)	0.631
Vitamin B12	311.5 (242–389)	–	–
Vitamin D	22 (16–27)	–	–
Folic acid	6.72 (4.8–8.75)	–	–
Total protein	73 (70–76)	–	–
Albumin	46 (44–48)	–	–
T4	1.36 (1.24–1.54)	–	–
TSH	2.36 (1.49–2.62)		
Calcium	9.69 (9.38–9.99)	–	–
Magnesium	2.07 (1.97–2.15)		

MDA: malondialdehyde; sTfR: serum transferrin receptor 1; CRP: C-reactive protein; PLT: platelet; MCV: mean corpuscular volume; BUN: blood urea nitrogen ALT: alanine transaminase; AST: aspartate aminotransferase; LDH: lactate dehydrogenase; TSH: thyroid stimulating hormone. Statistically significant p-values are presented in bold.

A correlation analysis was performed to evaluate the relationship between blood sTfR and MDA levels within each group. A significant positive correlation between blood sTfR and MDA levels was observed in both the patient (r=0.700) and control (r=0.599) groups (p<0.001). Additionally, a correlation test was performed to examine the relationship between the parameters found to be statistically significant between the two groups and other parameters. In the patient group, ESR levels showed a positive correlation with serum amyloid A (r=0.570) and C-reactive protein (CRP) (r=0.725); these relationships remained significant even after Bonferroni correction (p=0.012 and p<0.001, respectively). In contrast, after Bonferroni correction, no significant correlation was found between creatinine levels and other parameters in the patient group. In the control group, no significant correlation was found between ESR and creatinine levels and other parameters after Bonferroni correction.

The median Juvenile Arthritis Disease Activity Score-27 (JADAS-27) score of the patient group was 4 (3–6), and the median disease duration was 9 (9–12) months. No significant correlation was found between JADAS-27 scores or disease duration and sTfR and MDA levels. The clinical characteristics of the patient group and the sTfR and MDA levels according to the clinical characteristics are presented in Table 2.

**Table 2:** The clinical characteristics of the patient group and the soluble transferrin receptor and malondialdehyde levels according to the clinical characteristics.

Clinical characteristics JIA patients (n=30)		MDA	p	STfR	p
JIA subtypes n (%)	Oligoarticular JIA 13 (43.33)	10.47 (9.05–16.18)	0.552	0.58 (0.53–0.65)	0.273
	Polyarticular JIA 6 (20)	14.98 (11.16–17.44)		0.67 (0.61–0.76)	
	Enthesitis-related arthritis 5 (16.67)	11.04 (10.95–12.68)		0.57 (0.56–0.58)	
	Psoriatic arthritis 4 (13.33)	19.38 (10.7–36.74)		0.83 (0.62–1.47)	
	Systemic JIA 2 (6.67)	19.81 (9.45–30.17)		0.69 (0.5–0.87)	
Treatment n (%)	Adalimumab 12 (40)	11.1 (9.61–17.32)	0.655	0.58 (0.53–0.65)	0.156
	Methotrexate 9 (30)	10.26 (9.05–21.13)		0.55 (0.5–0.67)	
	Etanercept 9 (30)	14.13 (11.81–16.18)		0.71 (0.63–0.76)	
Treatment duration n (%)	6 months 22 (73.33)	13.4 (10.47–17.06)	0.830	0.62 (0.56–0.73)	0.830
	9 months 4 (13.33)	10.04 (8.26–29.94)		0.62 (0.48–1.33)	
	12 months 4 (13.33)	15.29 (8.34–25.65)		0.57 (0.53–0.73)	

MDA: malondialdehyde; sTfR: serum transferrin receptor 1; JIA: Juvenile idiopathic arthritis.

When evaluated in terms of JIA subtypes, the median MDA level was lowest in the Oligoarticular JIA subgroup and highest in the Systemic JIA subgroup. The median sTfR level was lowest in the Enthesitis-related arthritis subgroup and highest in the Psoriatic arthritis subgroup (Table 2).

In the treatment-based analysis, both MDA and sTfR levels were lowest in patients receiving Methotrexate treatment and highest in patients receiving Etanercept treatment. Regarding treatment duration, MDA levels were highest, while sTfR levels were lowest in patients receiving 12 months of treatment compared to other durations (Table 2).

However, there was no statistically significant difference in sTfR and MDA levels among subgroups created according to JIA subtypes, treatments applied, and treatment durations (Table 2).

## DISCUSSION

The most common rheumatic disease in childhood, and one that leads to significant long-term morbidity, is JIA, a collection of various adolescent chronic arthritis syndromes. Despite various studies, the prevalence and incidence of JIA are still unknown due to the lack of standard classification techniques and regional differences in disease frequency^10^. The epidemiological characteristics of the disease are crucial to determining how hereditary and environmental variables affect disease progression. Furthermore, it can improve preventive health practices and provide clues to the best treatment method. Each region has a different JIA prevalence^11^. Abnormal activation of T cells, B cells, natural killer cells, dendritic cells, macrophages, and neutrophils, and the production of pro-inflammatory mediators leading to joint destruction and systemic problems, are part of the pathophysiological stage of JIA^12^.

A recent meta-analysis examining 16 different studies reported that children with JIA have higher low-density lipoprotein cholesterol (LDL-C) levels compared to healthy children. Furthermore, it has been reported that dyslipidemia is more common in children with JIA than in healthy children, and that inflammatory conditions during the active phase of the disease or previous medication use may lead to lipid metabolism disorders in children with JIA^13^. Another study on lipid metabolism in children with JIA showed that dyslipidemia is common in JIA patients, particularly in the systemic subtype, and that this condition may be related to the underlying inflammatory process^14^. In contrast, MDA, a known biomarker of oxidative stress and end product of lipid peroxidation, is associated with chronic rheumatic diseases characterized by persistent inflammation, immune dysregulation, and progressive tissue damage; these processes are closely linked to increased ROS production. ROS contributes to synovial inflammation and to cartilage and bone destruction^15^. However, another recent study reported a positive correlation between MDA (a lipid peroxidation product) levels and LDL-C levels, and an association between MDA levels and dyslipidemia^16^.

In this study, no significant difference was found in MDA levels between children with JIA and their healthy peers. This may be due to the fact that most of the children with JIA included in the study had the oligoarticular subtype. Indeed, in the evaluation between subtypes, the highest MDA levels were found in children with systemic JIA, while the lowest levels were found in children with the oligoarticular subtype.

Iron storage disorders and/or low hemoglobin levels are frequently observed in individuals with rheumatic diseases, including JIA. The high prevalence of anemia and iron deficiency in this patient group suggests that dysregulation in iron metabolism may play a significant role in the pathophysiology of the disease^17^. A recent study reported that hepcidin levels, an important iron-regulating hormone, correlated positively with the JADAS-27 score in children with JIA and may be useful in the early diagnosis and monitoring of disease exacerbations^18^. High hepcidin levels lead to iron retention in macrophages and other cells; consequently, serum iron decreases, and functional iron deficiency anemia may develop^19^. Another recent study suggested that clinicians use biomarkers such as sTfR and hepcidin for a more accurate assessment of body iron status. However, hepcidin measurement is not yet universally accessible, and sufficient standardization among methods has not been achieved^20^.

In this study, no significant difference was found in sTfR levels between children with JIA and their healthy peers. Additionally, it was found that sTfR and MDA levels showed a positive correlation with each other, but no correlation with disease duration or JADAS-27 score. This may be related to the fact that the JIA children included in the study were under regular follow-up and did not have high disease activity. Indeed, the fact that the lowest sTfR levels were detected in JIA children who received the longest treatment also supports this view. Furthermore, metabolic reprogramming with methotrexate, commonly used in JIA patients, may mask or alter systemic ferroptosis biomarkers^21^.

Both iron deficiency and iron excess are harmful to human health, because iron excess can trigger ferroptosis-mediated cell formation by increasing the proliferation of ROS^20^. Ferroptosis, iron-induced cell death, is characterized by iron accumulation along with excessive lipid peroxidation^22^. In this context, the effects of methotrexate treatment on metabolic and oxidative pathways are of great importance. Indeed, a recent metabolomic study by Tomioka et al. showed that methotrexate created a different metabolomic profile in JIA patients and significantly altered pathways associated with oxidative stress, lipid metabolism, and amino acid metabolism^21^. Similarly, a study investigating iron status during anti-tumor necrosis factor (TNF) treatment in children with JIA reported that sTfR was useful in assessing iron deficiency in children with JIA and that sTfR levels returned to normal with anti-TNF treatment^23^.

In this study, both MDA and sTfR levels were found to be lowest in children with JIA who received methotrexate treatment. Additionally, other patients were receiving anti-TNF therapy. One reason for the lack of difference in MDA and sTfR levels between children with JIA and their healthy peers may be related to the treatment.

This study has some limitations. First, the relatively small sample size may have limited statistical power, particularly in subgroup analyses, and reduce the generalizability of the results. This may have increased the risk of Type II error due to the failure to detect some true differences. Second, the heterogeneous nature of the treatments administered in the patient group (treatment confounding effect) may have created a potential confounding effect at biomarker levels. Furthermore, it should be considered that the biomarkers evaluated have limited disease specificity and may be affected by different pathophysiological processes such as inflammation or oxidative stress. Therefore, the findings should be interpreted cautiously and supported by multicenter studies with larger, more homogeneous treatment groups. However, the preliminary data obtained in our study can be considered a strength, as they provide a reference and hypothesis-forming basis for future research with larger and more homogeneous sample sizes.

## CONCLUSION

In conclusion, although no systemic increase in sTfR and MDA levels was detected in this cohort followed under treatment, these findings do not rule out a possible role of ferroptosis in the pathophysiology of JIA. The absence of significant changes may be related to controlled disease activity and ongoing treatments. Therefore, the contribution of ferroptosis to JIA, especially in the context of specific disease stages or subtypes, remains an open question. Better-designed studies controlling for significant confounding factors such as methotrexate treatment are needed. Furthermore, given that sTfR and MDA are indirect markers, studies including glutathione peroxidase 4 (GPX4) activity measurement, comprehensive lipidomic analyses, and/or targeted tissue sampling (e.g., synovial fluid) are needed to allow for a more direct assessment of ferroptosis.

## Data Availability

The datasets generated and/or analyzed during the current study are available from the corresponding author upon reasonable request.
